# Osteo-Myelitis of the Long Bones

**Published:** 1880-02

**Authors:** 


### OSTEO-MYELITIS OF THE LONG BONES.

Dr. N. Senn, of Milwaukee, in an able article on spontaneous osteo-myelitis, published in the January number of the *Chicago Medical Journal and Examiner*, reaches the following conclusions:

1. Spontaneous osteo-myelitis is an infectious disease.
2. It is most prevalent in damp, changeable climates, and during the winter and spring months.
3. It affects with preference individuals during the period of growth and development of bone.

4. Traumatism and other agencies which produce a retardation or arrest of circulation in the vessels of the marrow act only as determining causes.

5. The primary seat is usually in the marrow of the cancellated tissue, in close proximity to the epiphysary cartilage.

6. Joint affections are frequent and prominent complications of this disease.

7. Thrombosis and inflammation of the veins of the marrow, bone, periosteum and soft parts are of frequent occurrence, and are the direct cause of pyæmia.

8. Swelling is absent for the first few days, and when it does occur, it becomes rapidly diffuse, and is attended by œdema and enlargement of the superficial veins.

9. Fluctuation is diffuse as soon as its existence can be ascertained.

10. A constant high temperature and typhoid symptoms indicate the gravest type of the disease.

11. Death may result from the intensity of the primary infection, but is usually produced by some complication.

12. Early removal of the products of inflammation under strictest antiseptic precautions, and local disinfection of the tissues are of paramount importance for its successful treatment.

13. Epiphyseolysis may become completely repaired.

14. Excision of shaft may become necessary, during the acute stage, to prevent exhaustion, from profuse suppuration ; this rule is not applicable if the humerus or femur is affected, on account of the impossibility of keeping the limb in position until regeneration of the bone has taken place.

15. In most cases, fixation of the limb is necessary for the purpose of procuring rest and to prevent deformity.

